# Fracture Risk of Long Bone Metastases: A Review of Current and New Decision-Making Tools for Prophylactic Surgery

**DOI:** 10.3390/cancers13153662

**Published:** 2021-07-21

**Authors:** Mỹ-Vân Nguyễn, Christophe Carlier, Christophe Nich, François Gouin, Vincent Crenn

**Affiliations:** 1 Orthopedics and Trauma Department, University Hospital Hôtel-Dieu, UHC of Nantes, 44000 Nantes, France; myvan.nguyen@chu-nantes.fr (M.-V.N.); christophe.carlier@chu-nantes.fr (C.C.); christophe.nich@chu-nantes.fr (C.N.); 2 PhyOs 1238, INSERM, University of Nantes, 44000 Nantes, France; 3 Leon Bérard Center, Surgery Department, 69008 Lyon, France; francois.gouin@lyon.unicancer.fr

**Keywords:** long bone metastases, pathologic fracture, impending fracture, skeletal-related events, predictive scoring system, metastatic bone disease

## Abstract

**Simple Summary:**

Long bone metastases are frequently a pivotal point in the oncological history of patients. Weakening of the bone results in pathologic fractures that not only compromise patient function but also their survival. Therefore, the main issue for tumor boards remains timely assessment of the risk of fracture, as this is a key consideration in providing preventive surgery while also avoiding overtreatment. As the Mirels scoring system takes into account both the radiological and the clinical criteria, it has been used worldwide since the 1990s. However, due to increasing concern regarding the lack of accuracy, new thresholds have been defined for the identification of impending fractures that require prophylactic surgery, on the basis of axial cortical involvement and biomechanical models involving quantitative computed tomography. The aim of this review is to establish a state-of-the-art of the risk assessment of long bone metastases fractures, from simple radiologic scores to more complex multidimensional bone models, in order to define new decision-making tools.

**Abstract:**

Long bone pathological fractures very much reflect bone metastases morbidity in many types of cancer. Bearing in mind that they not only compromise patient function but also survival, identifying impending fractures before the actual event is one of the main concerns for tumor boards. Indeed, timely prophylactic surgery has been demonstrated to increase patient quality of life as well as survival. However, early surgery for long bone metastases remains controversial as the current fracture risk assessment tools lack accuracy. This review first focuses on the gold standard Mirels rating system. It then explores other unique imaging thresholds such as axial or circumferential cortical involvement and the merits of nuclear imaging tools. To overcome the lack of specificity, other fracture prediction strategies have focused on biomechanical models based on quantitative computed tomography (CT): computed tomography rigidity analysis (CT-RA) and finite element analysis (CT-FEA). Despite their higher specificities in impending fracture assessment, their limited availability, along with a need for standardization, have limited their use in everyday practice. Currently, the prediction of long bone pathologic fractures is a multifactorial process. In this regard, machine learning could potentially be of value by taking into account clinical survival prediction as well as clinical and improved CT-RA/FEA data.

## 1. Introduction

Bone metastases are frequently a pivotal point in the oncological history of patients, as they lead to a variety of complications [1,2,3]. Collectively known as “skeletal-related events” (SREs): pain, hypercalcemia, medullar compression, and fractures are used to reflect bone metastases morbidity. Worldwide, more than 1.5 million people experience bone metastases, especially from breast, prostate, and lung cancer. Of these, 50% develop an SRE within the first two years following their diagnosis [3,4]. Pathologic fractures of the long bones are one of the most severe complications of such SREs. First, this type of SRE impairs patient autonomy and quality of life. Carter et al. reported a time trade-off equal to 0.89 for pathologic fractures in breast cancer [5]. Furthermore, SRE fractures lead to a higher mortality rate [6,7,8,9,10,11,12,13,14]. In the study by Saad et al., unadjusted model outcomes showed that pathologic fractures were significantly associated with decreased survival in breast cancer, multiple myeloma, and prostate cancer [9]. With advances in cancer therapy and improved prognosis, SRE management is becoming a critical topic.

Although bone metastases are generally detected during oncological follow-up, pathologic fractures are sometimes the first event leading to the diagnosis of a tumor. In addition, compared to preventive stabilization, surgical management of pathologic fractures results in an additional cost of USD 21,000 per patient, along with an average length of stay that is twice as long as usual [15,16]. Bone surgery, along with spinal cord decompression, has been reported to be the most expensive SRE treatment [5]. Moreover, prophylactic surgery procedures, such as curative resection and prosthetic reconstruction of impending femoral fractures, lead to better survival rates than osteosynthesis of actual pathologic fractures for some patients [17,18]. Along with arthroplasty, intramedullary nailing and plating with bone cement injections are part of the preventive surgical procedure in long bone metastasis management [19]. In light of this, timely identification of patients with a high risk of fracture is paramount [20,21,22,23].

Nevertheless, the indication for surgery depends on the patient’s overall prognosis, the localization of the lesion [24], and the inherent radiotherapy and chemotherapy delays. In addition, out of 1195 non-spinal skeletal metastases surgically treated in specialized sarcoma centers in Scandinavia, there was a 12.9% rate of complications such as lung embolism, liver failure, aspiration pneumonia, deep vein thrombosis, and fat embolism [18]. It is, therefore, recommended that long bone lesions that do not jeopardize the mechanical properties of the bone are treated conservatively. Accordingly, the main concern of tumor boards hence remains timely assessment of the risk of fracture, so as to provide preventive surgery while also avoiding overtreatment.

When surgery is not recommended, there are other treatment options. For example, external radiotherapy has been shown to be a valuable tool in MBD management, with one main objective: analgesic control [25], with effectiveness demonstrated not only for nociceptive pain but also for neuropathic bone pain [26]. According to the systematic review by Falkmer et al., there is strong evidence that radiotherapy of skeletal metastases provides overall pain relief in more than 80% of patients, lasting more than six months [27]. Moreover, bone recalcification can be expected after radiotherapy for multiple myeloma [28]. 

The data regarding pathologic fractures following radiotherapy are controversial: Sze et al. reported a 3.0% pathological fracture rate after single-fraction radiotherapy, and a 1.6% rate after multi-fraction therapy (*p* < 0.05) [29], whereas Chow et al. reported a 3.2% pathological fracture rate after single-fraction vs. a 2.8% rate after multi-fraction radiotherapy [30]. Nonetheless, it should be pointed out that radiotherapy in skeletal metastases is palliative in most cases, with relatively low-dose radiation. Indeed, in conventional external beam radiotherapy, various fraction regimens have been described, ranging from single-fraction (maximum 8 Gy) to short (maximum 22.5 Gy) or long-course multi-fraction radiotherapy (maximum 30 Gy) [31]. Consequently, considerations for late-onset fragility fractures after therapeutic dose radiation [32], which are much higher, cannot be applied in palliative radiotherapy MBD management. Similarly, non-union issues, as well as fractures due to embrittlement of the bone tissue, have only been described for high-dose radiation of up to 50 Gy, as used in soft tissue sarcoma treatment [33].

Furthermore, systemic treatments could also decrease the SRE rate. Of these, bisphosphonates such as zoledronic acid have been approved for the treatment of bone metastases, provided there is careful renal monitoring. By impairing osteoclast-mediated bone resorption, these entities affect osteoclast differentiation and their resorptive activity, thereby inducing apoptosis [34]. Denosumab, which is an antibody that inhibits RANK ligand is another new agent that affects osteoclast activity, thereby reducing the risk of SRE [35]. 

In this last decade, a combination of nuclear imaging medicine with radiopharmaceutical research has resulted in a new concept development called theranostics, enlarging the therapeutic arsenal for MBD. Relying on substitution of β+ by β- or α-particle emitters, the PET tracer is switched for a therapeutic radionuclide, exploiting the same specific molecular target for imaging and therapy [36]. In particular, prostate-specific membrane antigen (PSMA) has become an attractive target for radionuclide-chelating ligands [37]. As PSMA is over-expressed in prostatic cancer cells, the ^68^Ga PSMA-ligand was developed for positron emission tomography imaging. Combined with computed tomography (CT), ^68^Ga PSMA-11 PET/CT outperformed CT alone to detect bone metastases, with a sensitivity of 99% and specificity of 88%, while a CT scan showed a sensitivity and specificity of 87% and 61%, respectively [38]. It then can be used as a predictive biomarker to confirm target expression for endoradiotherapy, which can be achieved with 177Lu PSMA-617 β− emitters [39]. Similarly, since bisphosphonates demonstrated a high hydroxyapatite affinity along with the capacity to impair osteoclastic activity, it became a good candidate for theranostics. In this way, 68Ga-/177Lu-DOTA-ZOL seemed to be an effective palliative MBD treatment [40]. 

First described in 1989, the Mirels score assesses four prognostic items in the decision-making criteria for surgery [41]. It is presently the most widely used tool for this purpose, guiding surgical preventive management of pathologic fractures. Nevertheless, numerous studies have pointed out its lack of specificity, which often leads to overtreatment [22,42,43,44,45,46]. As a result, assessment based on a 30-mm axial cortical involvement (ACI) threshold has recently been described [46], with these new guidelines outperforming the Mirels score. Nonetheless, many authors report difficulty with predicting the occurrence of fractures in a context of metastatic bone extension. On the basis of mechanical models for fracture risk assessment, two approaches have been suggested. These are based on computed tomography and computational tools to model bone rigidity (computed tomography rigidity analysis, CT-RA) and bone strength (computed tomography finite element analysis, CT-FEA) [47]. The aim of this review was to describe the state-of-the-art in the risk assessment of long bone metastasis fractures, from the Mirels score and ACI to the recent CT-RA and CT-FEA mechanical models, as well as the progress that can be achieved with machine learning.

## 2. Fracture Risk Assessment in Long Bone Metastases: Standard Radiography and Nuclear Imaging Tools

Since the seventies, numerous authors have sought to identify impending fractures in association with long bone metastases [46]. Most of the studies to date, however, have lacked supportive statistical evidence. In the first report by Beals and Snell in 1964, 58% of the 19 femoral pathologic fractures in a breast cancer context were predictable. A 2.5-cm diameter lesion, either lytic or painful, was reported in each case. Seven years later, in their second report that took into account these results, half of the 10 impending fractures in 34 affected femurs were fixed [48]. In 1981, Fidler analyzed 100 pathologic fractures in long bones and concluded that they were likely to occur when more than 50% of the cortex was destroyed [49]. 

### 2.1. The Mirels Scoring System

#### 2.1.1. A Clinical–Radiological Composite Prognostic Score

In 1989, Mirels described a composite prognostic score based on four clinical and radiographic criteria: the lesion size (in relation to the bone diameter), its radiological appearance, its anatomical site, and its related pain. Each item is subdivided into three categories, rated from 1 to 3, resulting in a total score of between 4 and 12 [41] (Table 1). According to ROC curve analysis, maximum sensitivity and specificity are reached with scores equal to or higher than 9/12, related to a 33% risk of fracture, along with 0% false positives. Prophylactic surgery can thus reliably be proposed when diagnosing an impending fracture when there is the Mirels score is greater than 8. This predictive tool with a 9/12 cut-off score is hence considered the gold standard decision-making criterion for prophylactic surgery in cases of impending fractures of long bone metastases [41]. The fracture risk is deemed low in a given location with scores ≤ 7, moderate with a score = 8, and high with scores ≥ 9 (which indicates surgery).

#### 2.1.2. Limitations of the Mirels Scoring System

However, Mirels scores of 7 or 8/12 are related to 4% and 15% fracture risks, respectively, which can lead to undertreatment, although the false-positive rate increases (from 6% for a Mirels score = 8/12, up to 22% for a Mirels score = 7/12). A recent study comparing the Mirels score associated with prophylactic treatment of bone metastases along with actual fractures showed that the Mirels score tended to underestimate the risk fracture in upper limb metastases [50]. 

Furthermore, the score’s overall predictive value has been a matter of debate in the literature [51], since univariate analysis of each of its items has revealed a weak association with fracture risk [21,52,53]. In the series of Keen et al. with 516 metastatic breast lesions of the proximal femur, although the patients experienced moderate to severe bone pain, only 11% of them sustained fractures. Radiotherapy relieved the bone pain but did not preclude sustaining fractures, thus indicating that pain alone is not a reliable indicator of impending fractures [54]. Furthermore, the score exhibited low total inter- and intra-observer concordances (kappa = 0.294 and kappa = 0.323, respectively), with pain criteria associated with the highest inter-observer discordance [22,42,45]. In light of this, Howard et al. suggested adding a simple clinical criterion in conjunction with the Mirels score to predict pathological fracture. Using a threshold level of 85% of the single-leg stance weight-bearing capacity on the affected limb, they obtained a 0.97 sensitivity vs. 0.91 compared to Mirels scoring, a 0.65 specificity vs. 0.69, with a positive predictive value (PPV) of 0.78 vs. 0.79, and a negative predictive value (NPV) of 0.94 vs. 0.86 [55]. However, this criterion is limited to lower limb metastases assessment, as it depends on patient weight-bearing. Finally, the score’s lack of specificity, combined with a low PPV, may statistically induce overtreatments [42,46]. In the recent study by Van der Wal et al., a PPV of 14% indicated that six out of seven patients would undergo an unnecessary surgical procedure [56]. 

### 2.2. Axial Cortical Involvement (ACI) and Circumferential Cortical Involvement (CCI)

In 2003, on the basis of data from the Dutch Bone Metastasis Study Group, Van der Linden et al. suggested using a single objective radiographic criterion to assess fracture risk. They found that a cortical invasion of more than 30 mm in the axial plane (axial cortical involvement (ACI), ACI > 30 mm), detected on a frontal and lateral X-ray or CT scan, was more specific of the risk of fracture than the Mirels score [57]. Comparative data on the metrics of these two scores are presented in Table 2 [57]. A more recent prospective multicenter study has shown that the sensitivity, specificity, PPV, and NPV of axial cortical involvement for predicting femoral fractures were 86%, 50%, 20%, and 96%, respectively [56]. This fracture risk classification system has supplanted the Mirels score in the Netherlands and is now recommended for the management of long bone metastatic lesions [46]. Although it could be an improvement for establishing surgical indications, reducing excess surgery rates from 76% to 36%, it does present potential pitfalls. Its acceptable sensitivity (86%) associated with a specificity of only 50% and a PPV of 20% could lead to unnecessary prophylactic surgeries. Assuming that analysis of the dimensions of metastatic lesions could be insufficient on standard X-rays, Tatar et al. proposed use of three-dimensional CT scans in order to obtain a more precise assessment of cortical involvement. In their multivariate analysis, a circumferential involvement ≥30% was the only predictive parameter for pathological fracture [58]. 

### 2.3. Mirels Scoring System Applied to Scintigraphy 

Since 1989, improvements in technological engineering have led to considerable progress in nuclear medical imaging. Hybrid imaging technics combine anatomic data, from computed tomography (CT) or magnetic resonance imaging (MRI), with cross sectional details of functional information from radiotracers tumoral avidity [59]. As a result, prognostic assessments become more accurate, for early diagnosis as well as treatment follow-up of skeletal metastases [60].

Whole-body computed tomography, technetium-99m bone scintigraphy, and positron emission tomography–computed tomography (PET-CT) have become essential tools for assessing not only metastatic extensions of primary tumors but also the risk of bone metastasis fracture.

#### 2.3.1. ^99m^Tc MDP SPECT-CT 

In light of these technological developments, in 2018, Riaz et al. used technetium 99m-methyl diphosphonate (^99m^TC-MDP) single-photon emission computed tomography–computed tomography (SPECT-CT) scintigraphy as a substitute for radiography when evaluating Mirels scores [61]. Thirty-two cases were compared, using, on the one hand, a standard assessment based on Mirels scores and, on the other hand, SPECT-CT-modified Mirels scores. The changes mainly concerned the lesion’s size, with a distinction based on the axial size of the cortical invasion, as described by Van Der Linden et al. The results show a significant difference between the two scores (*p* < 0.01): 28% of the lesions were classified as being at high risk of fracture on the basis of the SPECT-CT-modified Mirels scores vs. 37.5% with Mirels scores based on X-rays. This pilot report combining hybrid bone imaging (X-rays along with SPECT-CT) has yielded promising results. The metabolic activity of the lesions was assessed, as high metabolic activity was considered to be high risk, whereas non-avid lesions were considered to be blastic lesions according to the Mirels scoring system. This novel hybrid score is an interesting add-on, and it may contribute to more precise prediction of the fracture risk in long bone skeletal metastases. Thus, it warrants being characterized in a larger prospective study. 

#### 2.3.2. ^18^F-FDG PET-CT 

Along these same lines, Ulaner et al. investigated whether 18F-fluorodeoxyglucose (^18^F-FDG) PET-CT quantitative measures of FDG avidity could be suitable for assessment of the femoral pathological fracture risk in patients with metastatic breast cancer. A total lesion glycolysis of 81 allowed identification of patients with a high risk of fracture. The sensitivity was reasonably good at 85%, the specificity was relatively high at 80%, the PPV was good at 67%, and the NPV was 91% [62]. Yet, ^18^F-FDG PET-CT is limited by its metabolic spectrum analysis, as it is not suitable for all types of cancers, and it also raises the question of accessibility of this decision-making tool in everyday practice. 

## 3. Biomechanical Models Based on Quantitative Computed Tomography

In light of the limitations of current imaging analysis practices, the need for a more accurate tool for predicting long bone fractures has shifted the focus towards biomechanical models.

In particular, quantitative computed tomography (QCT) has been centered on dimensions and mechanical analyses of metastatic osteolysis, using the finite element method (CT-FEA: computed tomography–finite element analysis) for modeling the stresses applied to the bone involved, or stiffness analysis (CT-RA: computed tomography–rigidity analysis). 

### 3.1. Computed Tomography–Rigidity Analysis (CT-RA)

#### 3.1.1. Modeling Bone Rigidity

The aim of CT-RA is to assess structural bone rigidity in all two-dimensional cross-sections of the bone, thereby helping to predict failure loads. In order to do this, X-ray attenuation (Hounsfield units) are converted to equivalent bone mineral ash density using a hydroxyapatite phantom included with each scan [63]. The relative position of each pixel to the centroid of the bone is recorded. Combining the spatial geometry information of each voxel element, as well as the bone density based on the grayscale level of the bone, CT-RA uses engineering composite beam theory to assess bone rigidity. In previous ex vivo studies, it was shown that the load-bearing capacity of the simulated lytic lesion was proportional to the rigidity at the “weakest” cross-section [64] and that CT-RA correlated well with yield loads of failure [65]. To calculate the actual bone strength, researchers compared the obtained rigidity data to the homologous cross-sections in the contralateral bone: a reduction in axial, bending, or torsional rigidities of more than 35% was taken to be an indication of a high risk for fractures [63,66,67].

#### 3.1.2. Assessment of Impending Fractures 

In the prospective multicenter study by Damron et al., 78 patients with femoral metastatic lesions were assigned Mirels scores, along with CT-RA assessments, and were followed up for 12 months until actual fracture, death, or survival without fracture. High-risk lesions according to CT-RA were associated with a 35% decrease in compression (axial), frontal and sagittal (bending), or rotational (torsion) stiffness. As a result, in this study, CT-RA provided higher sensitivity (100% vs. 66.7%), specificity (60.6% vs. 47.9%), PPV (17.6% vs. 9.8%), and NPV (100% vs. 94.4%) compared with the gold standard Mirels definition of impending fracture (≥9) [68]. In the study by Nazarian et al., 124 patients with 149 metastatic lesions were assigned to a treatment plan on the basis of the Mirels score. After obtaining the CT-RA results, the physicians changed the treatment for 36 patients. Seven of those who did not undergo fixation ended up suffering fractures [69]. Although all seven of these fractures were correctly predicted by CT-RA, the physicians were influenced by subjective criteria, considering pain and the primary source of metastasis before opting for preventive stabilization. Of note, only five of the seven lesions were predicted as being at high risk of fracture on the basis of the Mirels scores.

#### 3.1.3. A Step forward—Curved-Beam CT-RA 

This method was recently improved by taking into account the influence of intrinsic bone curvature. Indeed, as conventional CT-RA is based on straight beam theory, the resulting stress can be underestimated when the effect of curvature is not taken into account [70]. Whereas centroids of femoral shaft and neck sections correspond closely to straight lines, intertrochanteric regions should be approximated by curvature, in line with Wolff’s law, i.e., mechanical loads affect bone architecture [71]. As a consequence, a two-dimensional straight beam analysis in this site could not be of value, since mechanical stresses and loading forces in vivo are not restricted to a single plane [72]. In terms of three-dimensional curved beam theory, curved CT-RA appears to outperform conventional straight beam CT-RA. In this model, the iterative curved cross-sections were found when tangent vectors of curve passed through the centroid of cross-sections and perpendicular to them [70]. In the cadaver studies, curved beam CT-RA models predicted femoral failure loads for simulated lytic defects in strong correlation with the mechanical testing results. By contrast, straight beam CT-RA tended to overestimate failure loads in critical cross-sections, thereby implying potentially incorrect advice regarding weight-bearing recommendations. As long as one in five lesions occurs in the intertrochanteric site among femoral metastases [73], an accurate tool has to be validated in vivo in order to assess risk fracture in this particular area. 

### 3.2. Computed Tomography–Finite Element Analysis (CT-FEA)

#### 3.2.1. A Three-Dimensional Structural Modeling—Ex Vivo Studies

**Figure 1:**
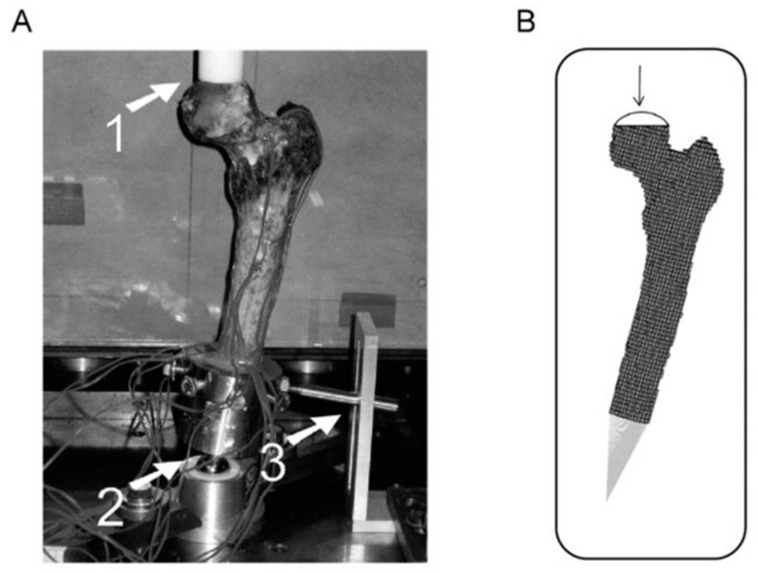
(**A**): Experimental setup and modeling using the CT-FEA method. (1) Plastic cup for applying the load; (2) point of rotation; (3) mechanism for locking the rotation. (**B**): Representation of the finite element model (From Tanck et al. [75], and Oftadeh et al. [70], CC-BY-SA 4.0).

This tool predicts femoral strength by means of subject-specific, three-dimensional structural modeling. As in CT-RA, a calibration phantom was used to approximate the Hounsfield units extracted from QCT to calcium-equivalent densities. Extracting elastic modulus, strength, and post-failure behavior data by quantitative computed tomography, finite element analysis (FEA) now relies on a large number of finite elements defined by density properties and bone geometry, thereby leading to the creation of a 3D computer model [74]. Several loading conditions can then be simulated, taking into account the patient’s weight along with the bone anatomy [66] (Figure 1).

##### Femoral Load-Bearing Strength in CT-FEA

Since the end of the 1980s, several studies have started to focus on FEA as a tool to predict fracture risk in metastatic lytic long bone lesions [76,77]. Initially, the elementary formulas simply accounted for the reduced cross-sectional area caused by a defect. McBroom et al. suggested measuring strength reduction in canine femurs with diaphyseal defects and to compare this with finite element predictions in tubular structures. They found that non-linear models, which accounted for material plasticity, provided a high degree of correlation with the experimental data. Given that bone fracture is in fact a non-linear phenomenon, higher correlations between the predicted and the measured failure load may be found using non-linear rather than linear FE models. Yet, this particular subject remains controversial [78,79,80]. At this stage, many trials have compared the load-bearing strength of cadaver bones to the critical strain predicted by FEA that leads to pathologic fracture. Using experimentally created lesions or cadaver bones containing metastatic lesions, the experimental loading was applied to the femoral head, and peritrochanteric, neck, or diaphysis lesions were evaluated. The model–experiment correlation varied from r^2^ = 0.77 to r^2^ = 0.98 [74,75,81,82]. 

##### The Femoral Inner Cortex Thickness Threshold

**Figure 2:**
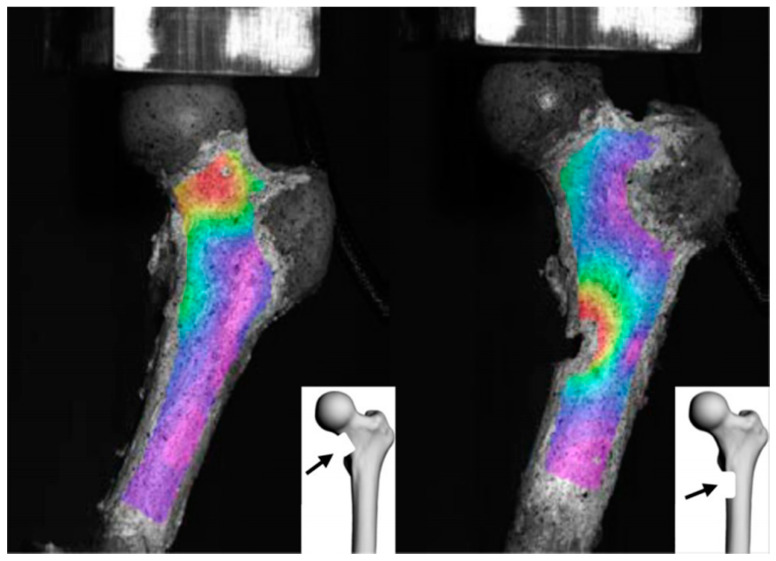
Von Mises strain analysis. Defect on the medial femoral neck (**left**), and under the lesser trochanter medial cortex (**right**) (from Riglet et al. [84], CC-BY-SA 4.0).

Interestingly, using femoral neck lesions created in cadaveric samples, Cheal et al. demonstrated that the greatest reduction in strength was predicted for the inferior-medial lesion, followed by the anterior lesion and then the superior-lateral lesion [77]. Similarly, Tanck et al. underlined that lesions located at the medial femoral side induce higher stress than anterior ones, irrespective of their size [75]. Indeed, under femoral head compression, both mechanical experiments and computer calculations revealed that the force is transferred mainly through the medial cortical bone of the femur. This may explain the greater loss of strength at the medial side under axial loading conditions. In light of this, using FEA on patient data, Kabawata et al. proposed a cut-off value of 3.67 mm for the thickness of the inner cortex as a predictor of pathological fracture, with a degree of high accuracy: 100% sensitivity and 75.1% specificity [83]. Hence, although the Mirels scores predicted that the anatomical site could be a matter of importance in assessing metastatic risk fractures, this outcome provides a greater degree of accuracy in the prediction of femoral pathologic fractures (Figure 2).

#### 3.2.2. Towards New Threshold Criteria: Strain Fold Ratio and Failure Load

CT-FEA was shown to outperform the Mirels score in a recent retrospective study including patients with femoral metastatic bone disease. Taking into account the patient’s weight, the femur anatomy, and a loading force representing stance position in their FEA modeling, Sternheim et al. calculated the strain fold ratio [85]. This value was defined as the ratio between the maximum principal strain in the vicinity of the tumor and the typical median strain in the contralateral region of healthy bones. Applying a 1.48 strain fold ratio as a predictive threshold for a pathological fracture, the FEA sensitivity was 100% compared with 88% for a Mirels score > 8, and the FEA specificity was 67%, compared with 38% for the Mirels score. It also revealed an area under the curve of 0.905 for SFR compared with 0.578 for the Mirels score (*p* = 0.008) [86].

Moreover, considering the currently used clinical guidelines in the Netherlands, i.e., axial cortical involvement, FEA models appear to be better at assessing fracture risk. In a prospective study simulating femoral failure loads in a sit-to-stand movement, normalized for body weight (BW) with a 7.5 × BW as a predictive cut-off for pathological fracture, the CT-FEA model had a 100% sensitivity (vs. 86% for ACI), a specificity of 74% (vs. 42%), a PPV of 39% (vs. 19%), and an NPV of 100% (vs. 95%) [87].

Furthermore, in a cadaveric study, Oftadeh et al. compared CT-FEA, CT-RA, and curved beam CT-RA for the quantification of failure loads and bone rigidity. The failure loads predicted by curved beam CT-RA and CT-FEA were in agreement with the experimental results. In addition, FEA yielded slightly more accurate estimates of the failure load than either of the two CT-RA models [70]. Given that CT-FEA may model complex loading conditions better than CT-RA, this result might be especially crucial for assessment of the risk of fracture in trochanteric lesions, given their relationship with muscle force attachment points. In particular, muscle loads transferred to the femur during daily living activities have to be considered. Muscles with large attachment areas cannot be approximated by concentrated forces applied through the centroids of their insertion. Indeed, FEA models with simplified load cases generated higher strain magnitudes than the physiological ones. This is of particular importance for the iliotibial tract and the gluteus medius role, as described by Pauwels, underlining that femoral stresses differ according to the muscles and ligament influence, particularly in the upper part of the femur compared to the diaphysis [53,88]. However, designing the entire muscle insertion significantly complicates the modeling and requires specific anatomical knowledge [89].

#### 3.2.3. FE Models: A Need for Global Standardization

Image-based FEA modeling has undergone development for more than 20 years. In order to predict bone strength and its subsequent risk of fracture, it integrates personalized mechanical failure determinants such as body weight, bone microstructure, and loading scenarios. Yet, variabilities in FEA modeling techniques have been reported, thus highlighting the need for standardization of scan acquisitions, load conditions, and outcome parameters [90].

##### Flattening the Inter-Scanner Differences in QCT Analysis

Firstly, although anthropomorphic standardization phantoms are commonly used, inter-scanner differences in QCT-based measurements of bone density and bone strength persist, which may limit its uptake [91]. Indeed, significant differences in grayscale values have been found depending on various parameters: slice thickness, power, and anthropomorphic or water phantom [92]. Studies with cadaveric bone tissue have been carried out with high amperage in order to increase image quality for FEA input. Conversely, clinical QCT scans are usually managed with a low tube current time to limit the amount of radiation to the patient. The strength and stiffness estimated from high-resolution scans have been reported to be greater than those obtained from low-resolution scans, except for the strength of normal and osteopenic femurs, which had lower values [93].

Additionally, regarding acquisition modalities, FE models have been shown to have a number of specific constraints. Indeed, arthroplasty implants can result in artefacts that affect the Hounsfield unit (HU) and corrupt the material characterization in CT-FEA models [85,87], thereby excluding patients with total hip or knee arthroplasty from FEA. Similarly, air artefacts around the phantom have been described, causing a degree of shading on the calibration phantom [87]. Eggermont et al. suggested overcoming this problem by using air–fat–muscle calibration extracted from a histogram of the HU in a standardized region of interest. They found no differences in failure loads between nonlinear FE models with phantom and air–fat–muscle calibration [94]. 

##### Standardized Modeling Constitutions in CT-FEA Modeling

Secondly, FEA studies have revealed variabilities in modeling constitution. Indeed, the choice of a specific density–elasticity relationship and the material-mapping method significantly influence FE outcomes [95]. In addition, the accuracy of the models is limited by the use of isotropic material properties. Yet, cortical bone is transversally isotropic, whereas trabecular bone is orthotropic [74]. Improvements in FEA modeling, integrating derived subject-specific anisotropic mechanical properties, have been shown to slightly enhance bone strength and stiffness predictions in pooled stance and side-fall configurations [96,97]. 

Moreover, loading conditions also participate in FEA study variabilities. Although linear FE models have been shown to provide good model–experiment correlation [80], the level of accuracy was insufficient to identify impending fractures during single-limb stance. Given that pathological hip fractures often occur during daily living activities without a fall [21], modeling of this particular configuration is needed. This way, non-linear models integrating post-yield behavior and crack propagation were found to improve fracture prediction in single-limb stance [79].

##### The Particular Case of Blastic Lesions in CT-FEA Modeling

In case of MBD, FE models accounting for the internal pressure exerted by a tumoral lesion have been shown to change the fracture load and mechanisms [98]. Indeed, mechanical changes occur in the tumor microenvironment due to cell mechano-sensitivity and cytoskeletal aberrations that lead to morphological cell alterations and remodeling of cell adhesion. Acidic and hypoxic conditions may impede bone structure reliability. In addition, the rapid proliferation of cancer cells gradually increases the pressure [99] and may affect the overall mechanical response of the femur so that the tumoral environment is more complex than an empty tissue. In particular, blastic lesions, which are a focal criterion in the Mirels scoring system, have been reported to affect bone density, elastic moduli, and femur strength properties [100,101]. Assuming that it could lead to overestimation of femoral bone strength, Sternheim et al. decided to exclude patients with predominantly blastic lesions from their study [86].

##### Selecting the Threshold Outcome Parameter in CT-FEA Modeling

Finally, consensus regarding threshold outcome parameters, along with a uniform image-based FEA methodology, are needed to provide a reliable risk of fracture assessment tool. Whereas Sternheim et al. used a critical SFR of 1.48 [85], Kawabata et al. proposed a threshold value of 3.67 mm for the thickness of the inner cortex [83]. Eggermont et al., on the other hand, focused on a 7.5 x BW cutoff point [102]. Goodheart et al. suggested combining FEA level walking + a Mirels score > 8, providing a sensitivity of 80% and 100% specificity [103]. Surprisingly, the level walking that they used was only equal to 2.5 x BW, which can be assumed to be the baseline load conditions on the hip joint [104]. Although parameters have to be harmonized, and there are limitations for blastic lesions, CT-FEA appears to yield the best predictive values for assessing long bone fracture risk (Table 3).

## 4. What Are the Next Steps?

### 4.1. Net Benefit Analysis: A Help for Surgical Indications in MBD?

As previously stated, preventive surgical procedures of long bone metastatic lesions depend on a benefit vs. harm balance analysis. Given that prognostic models based on measures of accuracy do not take into account clinical consequences, Vickers and Elkin sought to devise a method to evaluate prediction models by incorporating benefits and harmful consequences, which requires only the dataset on which the models have been tested [105]. This decision curve analysis model assumed that the probability of preferring a treatment relies on how the patient and the physician weigh up the relative harm of a false-positive and a false-negative prediction. By deriving a net benefit from these probabilities, some authors have recommended decision curve analysis as a method to be considered in preventive surgery for MBD [78]. However, as underscored by Kerr et al., this method is useful only if each patient has the same expected benefit and cost of intervention. Moreover, risk thresholds as predicted by net benefit curves are dictated by the risk prevalence [106]. Considering that the mechanical behavior of metastatic lesions of long bones can differ depending on the primary tumor, thereby resulting in distinct consolidation patterns after radiotherapy, a single scoring system for risk fracture assessment could be limited in terms of accuracy [107].

### 4.2. Machine Learning: Multimodal Data Management in a Single Decision-Making Tool

Currently, three-dimensional models outperform the Mirels score and the ACI Index for assessment of fracture risk, as mentioned above. Nevertheless, it needs to be kept in mind that imaging should not be the only basis for surgical decision-making: assessment of the risk of fracture should incorporate other contributing parameters, such as biological and clinical data.

Consequently, decision-making tools need to take into account not only radiological findings but also clinical, biological, and quantitative molecular imaging methods parameters, which may influence each other. In this context, machine learning (ML) is expanding in a wide range of medical applications. It has increasingly come to the forefront in recent years, partly owing to the advent of big data [108,109].

In machine-learning, neural network-based models of computation such as artificial neural network (ANN) enable a large number of computing elements (or artificial neurons) to connect and interact with each other. By allowing management of big non-structural data, it soon became the most popular type of algorithm used to model fast simulations of anatomical structures [110]. Alternatively, Bayesian belief network (BBN) modeling is based on conditional probabilistic relationships between variables. It helps in the interpretation of these structural data by estimating the likelihood of an outcome. The use of machine-learned BBN is increasing in various medical fields, such as helping to analyze complex clinical elements in order to provide clear guidelines as a decision-making tool.

#### 4.2.1. Real-Time FE Models Generation

As AAN deals with big non-structural data management, its relevance to improve biomechanical modeling based on quantitative computed tomography is clear. Even though FEA simulations were initially reported to take 8 h per sample for complex biomechanics analysis requirements [47,70,80], they can now be an automatic process and take only an hour to complete [86]. Similarly, future work focusing on machine learning-based approaches could help generate fast automated FE models of pathologic fractures in MBD. In this way, machine-learning models trained with data coming from finite element simulation of a side fall have already been demonstrated to provide an assessment of the risk of hip fracture in osteoporotic patients with a high degree of accuracy (87%) [111]. Furthermore, using machine-learning, FEA-based simulations for bone damage have been reduced from 1800 min to 1000 milliseconds in the past decade, thus allowing for considerable time savings [110,112].

#### 4.2.2. Predicting Survival after Bone Metastases Surgery

The contribution of orthopedic surgeons to preventing pathologic fractures in MBD management could help to decrease the incidence of this particular impairing skeletal-related event. However, given the potential post-operative complications, not all metastatic long bone lesions warrant being operated on. The patient’s general oncologic status and medical condition, and above all their life expectancy, are critical points that need to be evaluated before any surgery is undertaken. In this regard, machine-learned BBN models have been successfully developed and trained to estimate survival in patients undergoing surgery for bone metastases [113,114,115]. In PATHFx 3.0, various data are taken into account such as gender, the Eastern Cooperative Oncology Group (ECOG) performance status, and the number of bone metastases and visceral or lymph node metastases. Patients are followed for common types of solid cancers such as lung, hepato-cellular, sarcoma, breast, prostate, thyroid, and renal cell carcinomas, as well as myeloma and lymphoma. The AUC (area under the curve) for the PATHFx model is as high as 0.70 for predicting 3-month and 6-month survival. This tool could hence help physicians to opt for palliative radiotherapy rather than surgery for patients assessed as having a low chance of survival [115]. Although AAN appears to have better accuracy in predicting patient life expectancy after surgery for MBD (AUC = 0.89) [113], BBN may be easier to use since it can accommodate missing input data [116].

Finally, given that it is available online (www.pathfx.org, (accessed on 14 June 2021)), PATHFx could be a useful clinical decision support tool based on machine learning. In the survey by Cumming et al., only 5 out of 28 oncologists routinely used a fracture prediction score, thus highlighting the importance of a multidisciplinary approach in MBD management, with involvement of an orthopedic surgeon to advise regarding preventive stabilization of impending fracture [117]. This type of easy-to-use tool may be an impetus for healthcare providers to more widely employ prediction scores in clinical practice.

#### 4.2.3. Considering Both Radiological and Clinical Data at the Same Time

In 2017, Oh et al. focused on patients with lung cancer and femoral metastases. Using machine-learning algorithms to combine CT-RA data and clinical features, they demonstrated that including clinical criteria such as gender, osteolysis, and the absence of radiation therapy improved the power of pathologic fracture prediction compared to CT-based radiological features on their own [118]. Moreover, with the aim of SRE prediction in cancer patients with MBD, Wang et al. developed a machine-learning model based on a decision-tree that included four variables: a VAS (visual analog scale), PINP (amino-terminal pro-peptide of type I collagen), CA 15-3, and BALP (bone-specific alkaline phosphatase), providing good sensitivity (87.9%) as well as good specificity (83.3%) [119]. As this model is focused not only on fractures but also on the overall SRE, it demonstrated the importance of biological data adjunction.

Overall, a similar tool could be devised for fracture risk prediction in long bone metastatic disease. Similar to the FRAX or CatBoost examples of fracture risk assessment tools in osteoporotic bones [120,121], it should include both clinical data as well as radiological parameters regarding femoral neck bone mineral density. Given the promising time savings in CT-RA/FEA models generated with machine-learning progress, it should be easier to integrate these personalized biomechanical models into everyday practice. Combining it with patient survival prediction data, as well as the biological and clinical parameters, machine-learning could provide major advances in impending pathologic fracture assessment.

## 5. Conclusions

Altogether, these results demonstrate that prediction of long bone pathologic fracture remains a clinical challenge for tumor boards. In many cases, MBD has to be managed with conservative treatments, although some patients with impending fractures are likely to benefit from preventive surgery. However, disease specificities such as life expectancy or responses to irradiation or chemotherapies have underscored the difficulty of establishing a general yet accurate predictive tool for pathologic fracture risk assessment.

Although the Mirels score and ACI have yielded relatively disappointing outcomes due to low specificity and positive predictive values, due to the limited number of criteria, on the basis of plain radiographs, they nonetheless provide a readily available and simple-to-use threshold for surgical intervention guidance. As such, they remain the gold standard guidelines in most centers.

Conversely, promising QCT biomechanical models such as CT-RA and CT-FEA have better specificities than the Mirels rating system. By modeling bone strength and load-bearing scenarios that take into account patient weight and bone density at the lesion site, these powerful strategies fully shift the focus away from standard fracture risk assessment tools. Yet, the uptake of CT-RA PPV remains relatively low, and these strategies will only be adopted in routine clinical practice if a fast, highly automated procedure can be implemented.

In particular, the imaging acquisition duration and the standardization of each procedure have to be improved so that a widespread use of these techniques could be implemented into each MBD management. Since tumor boards face the question of prophylactic surgery for impending fractures in their everyday practice, efforts should focus on these predictive tools availability and comparative studies to ensure their exploitation.

Currently, predicting impending fractures in long bone metastases is a multifactorial problem, and there is a need to better define the contribution of mechanical and non-mechanical features to fracture risk. This calls for a multidisciplinary approach involving oncologists, orthopedic surgeons, radiologists, radiotherapists, and nuclear medicine physicians. Moreover, given that lesions are likely to progress, leading to repetitive assessments during the follow-up of oncology patients, predictive independent factors need to be easy to input, limited in number and complexity, and cost-effective. To date, none of the available tools combine all of these qualities.

Finally, by not only allowing integration of clinical, biological, and survival predictive data, but also radiological parameters, the expansion of machine-learning applications has yielded encouraging outcomes that herald better pathologic fracture risk prediction in the future. Combined with personalized mechanical failure determinants provided by CT-RA or CT-FEA models, it would constitute a powerful making-decision tool.

## Figures and Tables

**Table 1:** The Mirels scoring system. Adapted from [41].

Score	Site of Lesion	Size of Lesion	Nature of Lesion	Pain
1	Upper limb	<1/3 of cortex	Blastic	Mild
2	Lower limb	1/3–2/3 of cortex	Mixed	Moderate
3	Trochanteric region	>2/3 of cortex	Lytic	Functional

**Table 2:** Comparative intrinsic validity and diagnostic values of the Mirels score and axial cortical involvement (ACI), as described by the Dutch Bone Metastasis Study Group [46], and Circumferential Cortical Involvement (CCI) [58]. PPV: positive predictive value; NPV: negative predictive value.

Fracture Risk Assessment	Sensitivity	Specificity	PPV	NPV
Mirels score > 9 [46]	100%	13%	14%	94%
ACI > 30 mm [46]	86%	58%	23%	97%
CCI > 30% [58]	100%	89%	71%	100%

**Table 3:** Diagnostic values comparison. ^1^ Total lesion glycolysis > 81; ^2^ reduction > 35% in axial, bending, or torsional rigidities; ^3^ linear model, failure load < 7.5 × BW; ^4^ non-linear model, SFR > 1.48; RT: radiation therapy; MDB: metastatic bone disease; * carcinoma, myeloma, and lymphoma patients; † in breast cancer patients.

Predictive Tool	Population	Sensitivity (95% CI)	Specificity (95% CI)	PPV (95% CI)	NPV (95% CI)
Mirels score > 8					
Sternheim et al., 2020 [86]	Femur palliative RT (*n* = 41)	0.88 (0.47–0.99)	0.38 (0.47–0.99)	0.32 (0.19–0.59)	0.90 (0.55–1.00)
Damron et al., 2016 [68]	Femoral MBD * (*n* = 78)	0.67 (0.22–0.96)	0.48 (0.36–0.60)	0.10 (0.02–0.23)	0.94 (0.81–0.99)
Van der Wal et al., 2020 [56]	Femur palliative RT (*n* = 100)	0.77	0.45	0.17	0.93
ACI > 30 mm					
Eggermont et al., 2020 [87]	Femur palliative RT (*n* = 50)	0.86	0.42	0.19	0.95
Van der Wal et al., 2020 [56]	Femur palliative RT (*n* = 100)	0.86	0.50	0.20	0.96
^18^F-FDG PET CT ^1^					
Ulaner et al., 2017 [62]	Proximal femur fracture † (*n* = 27)	0.85 (0.65–0.96)	0.80 (0.67–0.90)	0.67 (0.48–0.82)	0.91 (0.80–0.98)
CT-RA ^2^					
Damron et al., 2016 [68]	Femoral MBD * (*n* = 78)	0.99 (0.54–1.00)	0.61 (0.48–0.82)	0.18 (0.07–0.35)	1.00 (0.92–1.00)
CT-FEA					
Eggermont et al., 2020 ^3^ [87]	Femur palliative RT (*n* = 50)	0.86	0.74	0.39	1.00
Sternheim et al., 2020 ^4^ [86]	Femur palliative RT (*n* = 41)	1.00 (0.66–1.00)	0.69 (0.45–0.84)	0.53 (0.28–0.77)	1.00 (0.79–1.00)

## Data Availability

Not applicable.
